# Missed opportunities for brief intervention in tobacco control in primary care: patients’ perspectives from primary health care settings in India

**DOI:** 10.1186/s12913-015-0714-6

**Published:** 2015-02-01

**Authors:** Rajmohan Panda, Divya Persai, Sudhir Venkatesan

**Affiliations:** Public Health Foundation of India, New Delhi, India; Division of Epidemiology and Public Health, University of Nottingham, Nottingham, UK

## Abstract

**Background:**

World Health Organization has called for tobacco cessation to be integrated into primary care. Primary Health Centres (PHC) offer opportunities for tobacco-use screening and brief cessation advice but data on such activities in developing countries such as India are limited. The aim of this study was to investigate screening and brief intervention practices of health service providers in primary care.

**Methods:**

This cross-sectional study was conducted in 2012 among 1,549 patients aged over 18 years visiting PHCs in 12 districts of two Indian states- Andhra Pradesh and Gujarat. Responses were collected using an interviewer-administered questionnaire. Information was obtained on participants’ tobacco use status, reason(s) for seeking medical care, whether participants had been screened for and advised to quit tobacco use. The primary outcome was whether patients were screened during their visit to the PHC. Data analysis was performed using multi-level logistic regression.

**Results:**

Less than one-third (447) of patients were screened for tobacco use during their visit to the PHC. People presenting with respiratory complaints were 84% more likely (OR: 1.84; 95% CI: 1.30 to 2.62) to be screened for tobacco use when compared to those with general ailments. Number of quit attempts in the past 12 months was strongly associated with the outcome of being screened for tobacco use, indicating that people who had more than 5 quit attempts were two times more likely to be screened for tobacco use than those who had never attempted to quit tobacco (OR: 1.99; 95% CI: 1.03 to 3.8). Among the 447 patients who were screened for tobacco use, only 136 reported to have been counselled and merely 67 patients received suggestions on ways to quit tobacco.

**Conclusion:**

Our results show that opportunities for screening and providing tobacco use cessation advice were largely missed by the health service providers. Our study suggests that there is an urgent need to incorporate tobacco cessation interventions as part of standard practice so that all patients are given an opportunity to be asked about their tobacco use and to be given advice and counselling to quit tobacco.

**Electronic supplementary material:**

The online version of this article (doi:10.1186/s12913-015-0714-6) contains supplementary material, which is available to authorized users.

## Background

Tobacco is the single most significant cause of preventable morbidity and mortality globally [1]. The health implications of tobacco use are well documented and include deaths attributable to active and passive smoking and the use of smokeless tobacco [2]. Tobacco use is ranked as the one of the major preventable risk factors for mortality and morbidity in India. Smoking alone currently accounts for more than 100,000 deaths in India [3]. Proven tobacco control measures such as raising taxes, smoke-free laws, public awareness, capacity building, alternate cropping, and banning advertising among others are necessary to reduce the overall consumption of tobacco [4]. The majority of tobacco-related deaths that can be prevented over the next 40 years will be among current smokers who can be persuaded to quit [5].

Tobacco use is an addiction and cessation can substantially reduce tobacco-related morbidity and mortality [6]. In India cessation acquires an essential strategic dimension as tobacco is used in several smoking and smokeless forms and users may quit one form in favour of another. Tobacco cessation services include a package of brief interventions and counselling services. In 2002, a network of 19 Tobacco Cessation Clinics (TCCs) was set up over a period of time in selected tertiary level centres to study the feasibility of establishing tobacco cessation services in the country. These tertiary centres in India were well received and short-term outcome of subjects seeking help was encouraging [7]. However the existing TCCs are not sufficiently equipped to take care of any population-based cessation program. To address this problem, provision was made to establish tobacco cessation facilities at the district hospital under the auspices of the National Tobacco Control Program (NTCP). The National Tobacco Control Program has been launched in 21 states and 42 districts in the country. NTCP measures at district level include capacity building of health service providers in tobacco control and strengthening information, education and communication activities [8].

World Health Organization has called for smoking cessation to be integrated into primary health care [9]. Primary Health Centre (PHC) is the most common setting for the provision of tobacco cessation advice [10]. Health Service Providers (HSPs) at this level of health care are well placed to use patient’s visit as an opportunity for providing screening and brief interventions in tobacco cessation. Brief intervention has been recommended as a best practice for the management of tobacco dependence in clinical settings. Brief interventions consist of five major components (the“5A’s”) which include *Asking* about tobacco use, *Advising* to quit, *Assess* willingness to quit, *Assist* in quitting and *Arrange* follow-up [11]. However, brief intervention practices of HSPs at PHCs are hardly documented in India and the current available literature suggests that HSPs are not intervening in their patients’ tobacco use habits [12]. Through the present study we tried to investigate whether practices in tobacco cessation are a part of routine clinical practices of HSPs. We undertook exit interviews with patients visiting health facilities providing primary care. Through this study we investigated the screening and brief intervention practices of HSPs in tobacco cessation. We also present here the factors influencing screening practices of HSPs for tobacco usage in patients attending health facilities providing primary care.

## Methods

### Study design and procedures

This cross-sectional study was conducted among 1,549 patients aged more than 18 years visiting health facilities providing primary care in 12 districts of two high tobacco burden states i.e. Andhra Pradesh (AP) and Gujarat in 2012 (AP: 6 districts, Gujarat: 6 districts). The states of Andhra Pradesh and Gujarat were selected because, in addition to being the highest producers of tobacco in India, they also have high prevalence of tobacco use [13]. The six districts in each state were purposively selected in consultation with state government officials. The districts which were not part of the National Tobacco Control Program were selected to avoid contamination with the distinguishing tobacco control measures offered as a part of NTCP districts. These districts represent different sub-geographical regions of each state and thus the study findings can be generalizable across the respective states.

The study was conducted in 200 health facilities providing primary care. These health facilities include primary health centres (64%), community health centres (18%), urban health centres (14%) and district hospitals (4%). The health facilities were chosen using systematic random sampling. All the health facilities providing primary care in the district were listed. The first health facility was selected at random and then every fifth health facility was selected for inclusion in the sample.

Primary health care facilities are the cornerstones of health services- a first port of call to a qualified doctor of the public sector for the sick and those who directly report or are referred for preventive, promotive and curative health care. Responses were collected by means of an interviewer administered questionnaire, which comprised five sections namely a) ‘Participant eligibility’, b) ‘Socio-demographic information’, c) ‘Tobacco use information’, d) ‘Tobacco counselling practices by healthcare providers’, and e) ‘Motivation to quit and tobacco cessation’.

A situational analysis and literature review was conducted which helped develop the questionnaire [14]. Prior formative research was done to determine themes of the questionnaire. The questionnaire was administered by trained interviewers hired from a survey agency. The questionnaire was validated and pilot tested and changes were made. The interviews were conducted in local language. The interviewers established good rapport with the respondents before administrating the questionnaire. To reduce the social desirability bias, respondents’ names were kept anonymous and confidentiality was maintained.

### Recruitment of respondents and consent

The prevalence of tobacco use in both the states is 29% (GATS India, 2010). With a design effect of 2 for a two stage sampling and based on a margin of error of 5%, a confidence level of 95% and a non- response rate of 10%, the sample size of 700 for each state was found to be adequate. To achieve the desired sample size 7 patients were taken from one health facility. The study participants were recruited through simple random sampling by selecting every third patient who registered to see the health service provider on each consulting day during the study period excluding weekends and public holidays. The participant eligibility was determined on the basis of whether the respondent was adult (more than 18 years), sought services from health service providers, and consumed tobacco in some or the other form. Data collection was done at suitable places near to the vicinity of health facilities away from the consultation rooms. Critically ill patients, those younger than 18 years, and those who did not give consent were excluded from the study. Self-help material which would help in tobacco cessation and appropriate compensation for the time were provided to the patients. Data was collected from January to March 2012.

### Measures

#### Independent variables

Socio-demographic variables included age, sex, location (rural/urban), religion, community, marital status, level of education, employment, and socio-economic status. Respondents’ highest level of education was measured according to four categories i.e. ‘uneducated’, ‘less than primary’, ‘primary but less than secondary’ and ‘secondary and above’. Socio-economic status was captured by ‘below’ and ‘above poverty line’ status. For rural areas the national poverty line is estimated at Rs. 816 per capita per month and Rs. 1,000 per capita per month in urban areas in Andhra Pradesh (2011-12) [15]. Religion was recorded as either ‘Hindu, Muslim, Christian, Sikh, Jain or others’. Caste was measured as the most socially disadvantaged group which include scheduled castes (SCs) and the scheduled tribes (STs) [16]. In addition, some castes such as “Other Backward Castes” (OBCs) were also included in the analyses.

Other independent variables included form of tobacco used (smoked or smokeless tobacco), number of quit attempts made by the respondent over the past twelve months (none, 1 to 5 attempts, >5 attempts), number of visits to the healthcare facility over the past twelve months (1 or 2 visits, 3 to 5 visits, 6 visits and greater), presenting illness of the respondent (broadly categorised as general ailments, respiratory complaints, obstetric and gynaecological related consultation, chronic diseases such as diabetes and hypertension) and whether the respondent saw a medical or a paramedical member of staff (Table 1).

**Table 1:** **Characteristics of Independent variables**

**Characteristics**	**Description & measurement**
**Age**	Self-reported in years
**Sex**	Self-reported (male or female)
**Marital Status**	Self-reported as either ‘never married’, ‘married or living together’, ‘Divorced/separated’ or ‘Widowed’
**Education**	Self-reported as either ‘Primary incomplete (Not completed class VII)’, ‘Primary Complete (Completed class VII)’, ‘Secondary Incomplete (Not completed class X)’, ‘Secondary Complete (Completed class X)’, ‘Higher Secondary complete (Completed class XII)’, ‘College/University complete (Degree / PG)’ or ‘Other’
**Caste**	Self-reported as either General, Scheduled Castes, Scheduled Tribe, Other Backward Caste, or other caste (Caste definitions as recognised by the Government of India)
**Poverty**	Self-reported; national poverty line is estimated at Rs. 816 per capita per month for rural areas and Rs. 1,000 per capita per month in urban areas
**Area of residence**	Rural and Urban
**Employment**	Self-reported employment status as either ‘employed or unemployed’ derived from responses to question B09 in the questionnaire (Additional file 1)
**Number of quit attempts in the past 12 months**	Self-reported, derived from responses to question C18 in the questionnaire (Additional file 1)
**Form of tobacco**	Self-reported as either smoking form of tobacco, smokeless form of tobacco or both.
**Presenting Illness**	Self-reported; General ailments include fever, wounds, diarrhoea etc; Respiratory complaints include cough, TB and other respiratory conditions; Obstetric & Gynaecological complaints include ANC, family planning and delivery, Chronic conditions include cardiovascular diseases, asthma, diabetes and cancers; and other includes conditions that did not fit any of the above categories. Derived from question D02 in the questionnaire (Additional file 1).
General Ailments	
Respiratory complaints	
Obstetric & Gynaecological	
Chronic conditions	
Others	
**Number of visits in the past 12 months**	Self-reported
**Healthcare provider seen**	Recorded on the basis of the designation and qualification of the HSP seen. Derived from question D03 in the questionnaire (Additional file 1).

#### Screening for tobacco use

Respondents were asked whether they had been screened for tobacco use by the following question: “Have you been asked about your tobacco consumption habit during today’s visit?” Those that answered “Yes, during this visit” were ascertained to be “Screened for tobacco use”. Respondents who answered “Not during this visit, but during an earlier visit” or “Never during any of the visits” were coded as “Not screened for tobacco use”.

#### Individual components of brief intervention

For those respondents for whom the brief intervention (counselling) was offered responses on individual components of the brief intervention were recorded. This included an estimate of the duration of counselling provided and also about specific questions asked/advice given by the HSP during the brief intervention. The responses was captured on information provided by HSPs on harmful effects of tobacco, benefits of quitting, HSPs inquiry on patients’ intention and willingness to quit, information on ways to quit, medications for quitting and follow-up at higher centres (Additional file 1).

### Data analysis

“Screened for tobacco” was our primary outcome. We performed multi-level logistic regression to examine factors that may affect “screening for tobacco”. Several demographic factors were included in the analyses including socio-demographic factors, previous quitting attempts, presenting illness, visits to the health facility and health service provider. Since the study was conducted in 12 districts, ‘district’ was included as a random intercept into a hierarchical model to account for clustering of data within each district. Unadjusted odds ratios (OR) and 95% Confidence Intervals (95% CI) were first estimated for each of the predictor variables and then the statistically significant associations (p-value <0.05) were then entered into a multivariable model to obtain adjusted OR and 95% CI. All statistical analyses were carried out using Stata 12.0 (StataCorp. [17]).

### Ethical approval

A written consent was taken from all the respondents before conducting the interviews. Prior permission from the state and district authorities was taken before conducting the survey in respective districts and states. Public Health Foundation of India (PHFI) Ethical Committee (IEC no 65/60) granted ethical clearance for this study to be carried out.

## Results

A total 1,569 patients visiting primary health centres were interviewed. The response rate of the study was 95%. Data on the outcome (screening) was largely complete with just over 2% (35 patients). Majority of the respondents were male (79%) and the median age of the respondents was 45 years (IQR 35 to 56). Less than half of the respondents (42%) were uneducated and only 10% were educated to secondary school level or above. More than two-third (65%) of respondents were recorded as being ‘below the poverty line’ and 76% of them resided in rural settings. Smokeless tobacco users account for a slightly greater proportion of study sample as compared to smokers (47% and 45% respectively). General ailments were the most common presenting conditions (51%) of patients at PHCs followed by respiratory complaints (30%). Table 2 portrays background characteristics of the patients. Missing data were assumed to be Missing Completely At Random (MCAR). Listwise deletion of the missing observations was performed and a complete-case analysis was undertaken. This method is robust even to violations of the MCAR assumption and known to produce unbiased and ‘honest’ estimates [18].

**Table 2:** **Background Characteristics of the participants**

**Characteristics**	**Total Number (%) (N = 1569)**	**Screened (n = 447)**	**Not screened (n = 1087)**
**Age** median (IQR) *in years*	45 (35 to 56)	41 (30 to 50)	44 (34 to 55)
**Sex**			
Female	321 (21)	87 (20)	230 (21)
Male	1,238 (79)	359 (80)	855 (79)
**Marital Status**			
Not married	282 (18)	81 (18)	196 (18)
Married	1,271 (82)	366 (82)	882 (82)
**Education**			
Uneducated	643 (42)	152 (35)	480 (45)
Less than primary	399 (26)	114 (26)	276 (26)
Primary but less than secondary	342 (22)	120 (27)	216 (20)
Secondary and above	145 (10)	52 (12)	91 (9)
**Caste**			
SC	372 (24)	106 (24)	261 (24)
ST	180 (12)	42 (10)	136 (13)
OBC	603 (39)	185 (42)	402 (38)
General	373 (24)	103 (24)	265 (25)
**Poverty**			
Above poverty line	537 (35)	179 (41)	348 (32)
Below poverty line	1,008 (65)	256 (59)	734 (68)
**Area of residence**			
Rural	1,113 (76)	304 (75)	786 (76)
Urban	348 (24)	101 (25)	242 (24)
**Employment**			
Unemployed	18 (1)	8 (2)	10 (1)
Employed	1,536 (99)	439 (98)	1,072 (99)
**Number of quit attempts in the past 12 months**			
0	840 (57)	208 (51)	628 (60)
1 to 5	547 (37)	183 (44)	359 (34)
>5	87 (6)	21 (5)	65 (6)
**Form of tobacco**			
Smoked tobacco	705 (45)	188 (42)	505 (46)
Smokeless tobacco	736 (47)	230 (51)	496 (46)
Both	114 (7)	29 (7)	85 (8)
**Presenting Illness**			
General Ailments	788 (51)	217 (49)	566 (52)
Respiratory complaints	465 (30)	142 (32)	321 (30)
Obstetric & Gynaecological	48 (3)	20 (4)	28 (3)
Chronic conditions	59 (4)	10 (2)	49 (4)
Others	179 (12)	58 (13)	120 (11)
**Number of visits in the past 12 months**			
1 or 2 times	597 (40)	146 (34)	448 (42)
3 to 5 times	616 (41)	180 (42)	433 (40)
6 times or more	291 (19)	100 (24)	189 (18)
**Healthcare provider seen**			
Physician	1,086 (71)	337 (76)	748 (69)
Non-physician	440 (29)	106 (24)	332 (31)

Percentages presented in this table are column percentages.

Where numbers do not add up to 1569, data are missing.

### Screening for tobacco use

Less than one-third (447) of patients were screened for tobacco use during their visit to the health facility. A total of 27% (87 individuals) of females and 28% (366 individuals) of males were screened for tobacco use. The proportion of patients being screened for tobacco use increased with an increase in level of education– 24% (152 individuals) of ‘Uneducated’; 29% (114 individuals) of those with ‘Less than primary’ education; 35% (120 individuals) with ‘Primary but less than secondary’ education, and 36% (52 individuals) with ‘Secondary and above’ education. 27% (188 individuals) of smokers, when compared to 31% (230 individuals) of smokeless tobacco users, were screened.

Results from multi-level logistic regression analysis indicated that the ‘General’ caste were associated with a 40% decreased odds of being screened for tobacco use (OR: 0.60; 95% CI: 0.40 to 0.88) when compared to ‘Scheduled Castes’ (SC) (Table 3).

**Table 3:** **Predictors of tobacco screening**

**Variable**	**Screened for tobacco use**	
	**Unadjusted OR (95% CI)**	**Adjusted OR (95% CI)**
**Age**	1.00 (0.99 to 1.01)	NA
**Sex**		
Female	1	NA
Male	1.16 (0.86 to 1.56)	
**Marital Status**		
Not married	1	NA
Married	0.97 (0.71 to 1.33)	
**Education**		
Uneducated	1	NA
Less than primary	1.07 (0.78 to 1.47)	
Primary but less than secondary	1.25 (0.90 to 1.74)	
Secondary and above	1.22 (0.79 to 1.89)	
**Caste**		
SC	1	1
ST	0.92 (0.57 to 1.49)	0.84 (0.50 to 1.40)
OBC	0.74 (0.54 to 1.02)	0.71 (0.50 to 1.00)
General	0.68 (0.47 to 0.97)*	*0.60 (0.40 to 0.88)* *
**Poverty**		
Above poverty line	1	NA
Below poverty line	0.87 (0.67 to 1.14)	
**Area of residence**		
Rural	1	NA
Urban	1.12 (083 to 1.53)	
**Employment**		
Unemployed	1	NA
Employed	0.47 (0.16 to 1.32)	
**Number of quit attempts in the past 12 months**		
0	1	1
1 to 5	*1.46 (1.12 to 1.90)* **	*1.54 (1.16 to 2.05)* **
>5	*1.89 (1.03 to 3.47)* *	*1.99 (1.03 to 3.85)* *
**Form of tobacco**		
Smoked tobacco	1	1
Smokeless tobacco	*0.76 (0.58 to 0.99)* *	1.08 (0.79 to 1.48)
Both	0.93 (0.56 to 1.56)	0.92 (0.53 to 1.58)
**Presenting Illness**		
General Ailments	1	1
Respiratory complaints	*2.16 (1.58 to 2.95)* ***	*1.84 (1.30 to 2.62)* ***
Obstetric & Gynaecological	1.42 (0.76 to 2.66)	1.43 (0.70 to 2.90)
Chronic conditions	1.30 (0.59 to 2.89)	1.05 (0.44 to 2.52)
Others	1.20 (0.82 to 1.76)	0.97 (0.63 to 1.50)
**Number of visits in the past 12 months**		
1 or 2 times	1	1
3 to 5 times	*1.43 (1.07 to 1.90)* *	1.25 (0.92 to 1.71)
6 times or more	*1.74 (1.23 to 2.46)* **	1.39 (0.94 to 2.05)
**Healthcare provider seen**		
Physician	1	1
Non-physician	*0.49 (0.35 to 0.67)* ***	*0.48 (0.34 to 0.68)* ***

OR = Odds Ratio; 95% CI = 95% Confidence Intervals; *p < 0.05; **p ≤ 0.01; ***p ≤ 0.001.

‘Number of quit attempts in the past 12 months’ was strongly associated with the outcome of being screened for tobacco use. Patients who had made ‘1 to 5 quit attempts’ and ‘>5 quit attempts’ were associated with an OR of 1.54 (95% CI: 1.16 to 2.05) and 1.99 (95% CI: 1.03 to 3.85) respectively of being screened for tobacco use than those who had never attempted to quit tobacco. Smokeless tobacco users were associated with a decreased likelihood of being screened as compared to smokers [unadjusted OR of 0.76 (95% CI: 0.58 to 0.99)]. However, this association did not remain statistically significant in the multivariable model. The odds of patients with respiratory complaints being screened for tobacco use were 1.84 times (95% CI: 1.30 to 2.62) greater than for patients presenting with general ailments. Interestingly, an increase in the number of visits to the PHC in the past 12 months were associated with an increased likelihood of being screened for tobacco use in the unadjusted analyses, this however proved to be statistically non-significant in the multivariable analysis. Pharmacists were less likely (OR: 0.48; 95% CI: 0.34 to 0.68) to screen patients for tobacco use when compared to physicians. Model fit was assessed using the Hosmer-Lemeshow test and Cochran C-statistic (ROC area). Hosmer-Lemeshow chi-squared statistic of 3.56 (p-value: 0.89) and C-statistic of 0.773 (95% CI: 0.747 to 0.799) was obtained suggesting a good model fit.

### Counselling practices of HSP

Out of the 447 patients who were screened for tobacco use, only 136 (36%) reported to have been counselled. Out the 136 patients who received counselling, 113 (83%) and 109 (80%) patients were informed of the “harmful effects of tobacco” and the “benefits of quitting” respectively, merely 67 (50%) received suggestions on the “ways to quit”, 13 (10%) received information on the pharmacological methods of quitting and 22 (16%) were informed of “follow-up at higher centres”. Findings on other components of the brief intervention have been presented in Table 4.

**Table 4:** **Counselling practices of health service providers in tobacco cessation**

**Counselling practices**	**n (% of counselled patients) (N = 136)**
If you were offered counselling, how long did it last?	
<30 seconds	20 (14.7%)
30 seconds to 1 minute	43 (31.6%)
1 to 5 minutes	60 (44.1%)
>5 minutes	12 (8.8%)
Did the HSP inform about harmful effects of tobacco?	
Yes	113 (83.1%)
No	24 (16.9%)
Did the HSP inform about benefits of quitting?	
Yes	109 (80.2%)
No	1460 (17.5%)
Did the HSP ask about your intention/ interest to quit?	
Yes	74 (54.4%)
No	62 (45.6%)
Did the HSP asked for your willingness to quit?	
Yes	74 (54.8%)
No	61 (45.2%)
Did the HSP suggest ways to quit tobacco use?	
Yes	67 (49.6%)
No	68 (50%)
Did the HSP inform you about different medicines for quitting?	
Yes	16 (11.8)
No	120 (88.2)
Did the HSP informed you about the further follow up at higher centres?	
Yes	13 (9.6%)
No	123 (90.4%)
Did the HSP tell you when to return for follow-up counselling?	
Yes	22 (16.3%)
No	113 (83.7%)
Did the HSP explain your present health condition as a result of your tobacco use?	
Yes	72 (53.3%)
No	63 (46.6%)

## Discussion

Provision of advice and support to tobacco users by health service providers in primary care settings improves cessation rates [19]. To the best of our knowledge, this study is the first study documenting patients’ report of health service provider’s behaviour regarding tobacco cessation activities in primary health care setting in India.

Studies from developed countries revealed that tobacco cessation practices among adults aged 25–44 years were less prevalent than among older patients [20]. However, we did not find age to be associated with screening practices of HSPs. Our study suggested that tobacco screening practices of HSPs differ markedly by presenting diagnosis as patient suffering from respiratory diseases were more likely to be screened for tobacco use as compared to chronic diseases. Similar observations were made in another study conducted by Nawi Ng et al. in Indonesia in 2007 [21]. It should be recognized that primary care, endorsing the concept of patient centeredness, can offer opportunity for tobacco cessation to patients suffering from chronic diseases who visit public health centres regularly for check-ups, collection and renewal of medications.

Tobacco dependence is recognised as a chronic disease that requires ongoing repeated interventions and multiple attempts to quit [22]. Studies reveal that tobacco users who had attempted to quit previously were more likely to receive quit advice [23,24]. In line with the literature, our study indicates that patients who made previous quit attempts were more likely to be screened for tobacco use. Studies suggest that receipt of quit advice by multiple health professionals increased quit attempts and quitting [25,26]. A large number of health professionals offering cessation advice could substantially improve the cumulative effect on reducing tobacco use [27]. Thus, HSPs should provide consistent support to tobacco users with repeated quit attempts and anticipate challenges to quitting by arranging a timely follow-up visit. Behaviour modification for tobacco cessation by HSPs should also be considered and patients should be educated on withdrawal symptoms and relapse.

Our findings indicate that only a few patients visiting health facility were advised by health service providers to quit tobacco use. This is consistent to a study done by Omole et al. in South Africa in 2008 in which 12% of tobacco users receive tobacco cessation advice [28]. However, percentages reported in our study is less than the averages reported in GATS India 2009-10 data which depicts that among the tobacco users who visited health facility 52% and 71% of smokers were advised by health service providers to quit tobacco in the state of Gujarat and AP respectively [4]. When patients attend primary health facilities, an enquiry about tobacco exposure by a physician and brief advice to quit can increase the rates of tobacco cessation. Unfortunately, these opportunities were largely missed by HSPs in our study.

In the present study we found that physicians were more likely to screen patients for tobacco use as compared to pharmacists. Similar findings were observed in a study done by Tremblay et al. in Canada in which general practitioners were more involved in practices pertaining to screening of tobacco amongst patients as compared to other health professionals [29]. Research has indicated that tobacco cessation interventions applied by other health care staff may equally be effective in primary health care [27]. However, our study is not in line with the existing literature and more qualitative research is needed to identify practices of physicians and pharmacists in tobacco use screening.

Our findings suggest that only a few patients were informed about harmful health effects of tobacco, assessed for intention to quit and assisted with ways to quit and follow-up counselling. The lack of HSPs engagement in tobacco cessation interventions in the present study is similar to that observed in other developing countries such as Indonesia [19]. However, the results of our study on brief interventions practices of HSPs is contrary to what pertains in other countries such as Australia, the USA and China, where health service providers are reported to be more engaged in tobacco cessation interventions [30,31]. Our findings indicate an urgent need to integrate and strengthen delivery of tobacco cessation services into routine services of HSPs.

Several strategies have shown to enhance the quit attempts among tobacco users, including provision of pharmacotherapy. Use of pharmacotherapy increases quit rates 1.5- to 2.5-fold, and is a potentially valuable adjunct to any advice provided [32]. Our findings indicate that only a few HSPs provided information on medicines pertaining to tobacco cessation to the patients. This might be related to insufficient knowledge of HSPs on pharmacotherapy in tobacco cessation and/or its non-availability and uptake at primary health centres in India.

The present study is limited by the fact that it relied on self-reported responses of patients and is subjected to response and recall bias. Further, these results do not reflect the perspectives of health service providers or notations from the medical record. The cross-sectional design of the study was another limitation. Causality cannot be inferred based on the cross-sectional design. Majority of the respondents in our study were males and the study was conducted in selected public health facilities thus the generalizability of the results is limited to similar subpopulations visiting health facilities in India and may not be applicable to general populations. The inadequate presentation of females to the public health facilities can be related to the social norms and lack of acknowledgement of women health as priority [33]. Only 9% of patients were offered tobacco cessation counselling during the current visit. Due to lack of statistical power, a multi-level logistic regression analysis was not performed to find out associations between different components of brief interventions by HSPs and their screening practices. Instead, percentages of patients’ responses to each of the brief intervention-related questions have been presented.

## Conclusions

Our study indicates low engagement of health service providers in providing brief interventions in tobacco cessation in the two states of India. The results show that opportunities for screening and providing tobacco use cessation advice were largely missed by health service providers. The present study suggests that there is an urgent need to incorporate tobacco cessation interventions as part of standard practice so that all patients are given an opportunity to be asked about their tobacco use and to be given advice and/or counselling to quit along with reinforcement and follow-up. The information provided by this study can guide the development of targeted intervention programs in tobacco cessation in India and other developing countries.

## Additional file

Additional file 1:
**Interview schedule.**

## Abbreviations

PHC: Primary health centres
HSPs: Health service providers
