# Supplementary material for: Missed opportunities for brief intervention in tobacco control in primary care: patients’ perspectives from primary health care settings in India
Source: BMC Health Serv Res. 2015 Feb 1;15:50. doi: 10.1186/s12913-015-0714-6 (PMC4318137; doi:10.1186/s12913-015-0714-6)
Supplement: Additional file 1: — Interview schedule. [file 12913_2015_714_MOESM1_ESM.pdf]

DLHC Code | | |

Sl. No. | | | |

D	D	M	M	Y	Y

**Start time**	:
**End time**	:

Interview Schedule

EXIT INTERVIEW OF CLIENTS OF PUBLIC HEALTH FACILITY IN STEPS HEALTH SYSTEM INTERVENTION AREA

Facility Name:		
Facility Location	Public Facility Type	CODE
--	DISTRICT/SUB-DISTRICT HOSPITAL	1
--	CHC	2
Mandal---	PHC	3
District---	UHC/UHP/UFWC	4

SCREENING QUESTIONNAIRE

Q. No.	Question	Response(s)	Code	Remark<br>DK=99, NR=999
**SECTION A: PARTICIPANT ELIGIBILITY QUESTIONNAIRE**<br>**సెక్షన్ ఎ : పాల్గొనేవారి అర్హతను నిర్ణయించే ప్రశ్నాపత్రము**				
A1.	Did you see a health care service provider today?<br>మీరు ఈరోజున ఆరోగ్య సంరక్షణ సేవలనందించేవారిని చూసినారా?	Yes అవును	1	
		No లేదు	2	END INTERVIEW<br>**ఇంటర్వ్యూ ముగించుము**
A2.	Do you use tobacco in any form?<br>**మీరు ఏదైనా రూపంలో పొగాకు వాడుతారా?**<br>**(currently means tobacco use during last 12 months)**<br>**(ప్రస్తుతం అంటే గత 12 నెలల కాలంలో పొగాకు వాడకం గురించి)**	Yes, currently<br>అవును, ప్రస్తుతం	1	
		Not currently<br>ప్రస్తుతం కాదు	2	END INTERVIEW<br>**ఇంటర్వ్యూ ముగించుము**
		Never ఎన్నడూ లేదు	3	END INTERVIEW<br>**ఇంటర్వ్యూ ముగించుము**
A3	How old are you?<br>మీ వయస్సు ఎంత?	Age in completed years<br>పూర్తి అయిన సం॥లలో వయస్సు		END INTERVIEW If CLIENT IS < 8 years<br>**క్లైంట్ 8 సం॥ల లోపు అయితే ఇంటర్వ్యూ ముగించుము**<br>If CLIENT IS ≥ 18 YEARS » Section B<br>క్లైంట్ 18 సం॥ల కంటే ఎక్కువ అయితే సెక్షన్ బి కి వెళ్ళండి
A4	If client is minor (8 - 17 years old)<br>Are you accompanied by parents/ guardian?<br>క్లైంట్ మైనర్ అయితే (8–17 సం॥ల వయస్సు వారు అయితే)<br>మీతో పాటు తల్లిదండ్రులు / సంరక్షకులు ఉన్నారా?	Yes అవును	1	
		No లేదు	2	END INTERVIEW<br>**ఇంటర్వ్యూ ముగించుము**

--***FOR OFFICE USE ONLY***--

Interviewer…………………………………………………………………………………
Interview Result (encircle one)<br>□ Completed (1) □ Incomplete (2)<br>□ Refused (3) □ Not Eligible (4) □ Other (specify) (5)……………………………………………
Supervisor……………………………………………………………………………………………………
Keyed By……………………………………………………………………… Verified by…………………………………………………………………………………

Q. No.	Question	Response(s)	Code	Remark<br>DK=99, NR=999
**Section B1: SOCIO-DEMOGRAPHIC INFORMATION**<br>సెక్షన్ బి 1 : సామాజిక – వ్యక్తిగత సమాచారం				
B01	Sex (from Observation)<br>లింగము (గమనించినదానిని బట్టి)	Male మగ	1	
		Female ఆడ	2	
B02	What is the highest level of education you have completed?<br><br>మీరు పూర్తి చేసిన అత్యున్నత విద్యాస్థాయి ఏమిటి?	Illiterate/No Education<br>నిరక్షరాస్యుడు / చదువు లేదు	1	
		Primary incomplete (Not completed class VII)<br>ప్రాథమిక స్థాయి పూర్తి కాలేదు	2	
		Primary Complete (Completed class VII)<br>ప్రాథమిక స్థాయి పూర్తి అయినది	3	
		Secondary Incomplete (Not completed class X)<br>సెకండరీ స్థాయి పూర్తి కాలేదు	4	
		Secondary Complete (Completed class X)<br>సెకండరీ స్థాయి పూర్తి అయినది	5	
		Higher Secondary complete (Completed class XII)<br>ఉన్నత సెకండరీ స్థాయి పూర్తి అయినది	6	
		College/University complete (Degree / PG)<br>కాలేజ్ / యూనివర్సిటీ పూర్తి అయినది	7	
		Other (specify)<br>మరేదైనా (తెలుపండి)	8	
B03	What is your current marital status?<br>మీ ప్రస్తుత వైవాహిక స్థితి ఏమిటి?	Never married పెళ్ళి కాలేదు	1	
		Married or living together<br>పెళ్ళి అయినది లేక కలిసి జీవిస్తున్నారు	2	
		Divorced/Separated<br>విడాకులు తీసుకొన్నారు / విడిపోయినారు	3	
		Widowed భర్త / బార్య చనిపోయినారు	4	
B04	What is your religion?<br>మీ మతం ఏమిటి?	Hindu హిందూ	1	
		Muslim ముస్లిమ్	2	
		Christian క్రైస్తవ	3	
		Sikh సిక్కు	4	
		Jain జైన	5	
		Buddhism బౌద్ధ	6	
		Other (specify)<br>మరేదైనా (తెలుపండి)	7	
B05	Which community do you belong to?<br>మీరు ఏ కమ్యూనిటీకి చెందినవారు?	SC షెడ్యూల్డ్ కులం	1	
		ST షెడ్యూల్డ్ తెగ	2	
		OBC ఇతర వెనుకబడిన కులం	3	
		General జనరల్	4	
		Other (specify)<br>మరేదైనా (తెలుపండి)	5	
B06	Where do you live?<br>మీరు ఎక్కడ నివసిస్తారు?	Rural Area గ్రామీణ ప్రాంతం	1	
		Urban/Semi-Urban Area<br>పట్టణ / సెమీ అర్బన్ ప్రాంతం	2	
		Slum Area మురికి వాడ	3	
B07	Which of the following best describes your (your family) economic status at present?<br>ప్రస్తుతం మీ (మీ కుటుంబము) ఆర్థిక స్థితిని వీటిలో ఏది బాగా తెలుపుతుంది?	Above Poverty Line దారిద్ర్య రేఖకి ఎగువన	1	S » B09
		Below Poverty Line దారిద్ర్య రేఖకి దిగువన	2	
B08	Do you (your family) hold a BPL card (White card)?<br>మీకు (మీ కుటుంబము)నకు బిపియల్ కార్డు ఉన్నదా? (తెల్లకార్డు)	Yes అవును	1	
		No లేదు	2	

Q. No.	Question	Response(s)	Code	Remark<br>DK=99, NR=999
B09	Which of the following best describes your main work status over the past 12 months?<br>గత 12 నెలల్లో మీ ప్రధాన పని స్థితిని వీటిలో ఏది బాగా తెలుపుతుంది?	Labourer - Agricultural<br>కూలివారు – వ్యవసాయం	1	
		Labourer Non-Agricultural<br>కూలివారు – వ్యవసాయేతర	2	
		Self Employed - Agricultural<br>స్వయం ఉపాది గలవారు – వ్యవసాయం	3	
		Self Employed -Non-Agricultural<br>స్వయం ఉపాది గలవారు – వ్యవసాయేతర	4	
		Salaried – Government<br>జీతం వచ్చేవారు – ప్రభుత్వం	5	
		Salaried – Private జీతం వచ్చేవారు – ప్రైవేట్	6	
		Student స్టూడెంట్	7	
		Housewife గృహిణి	8	
		Unemployed నిరుద్యోగి	9	
		Retired రిటైర్ అయినారు	10	
		Others (Specify) మరేదైనా (తెలుపండి)	11	
B10	Which of the following describes your exposure to different media over the past 12 months? (Multiple response possible)<br>For YES CODE=1 and NO CODE =2<br><br>గడిచిన 12 నెలల్లో మీరు చూసిన / విన్న విభిన్న మీడియాలను వీటిలో ఏది బాగా తెలుపుతుంది?<br>(అనేక జవాబులు రావచ్చును)<br>అవును అన్న వాటికి కోడ్ =1 మరియు లేదు అంటే కోడ్=2	Reads a newspaper/magazine at least once a week (Skip if client is illiterate)<br>వారానికి కనీసం ఒకసారి దినపత్రిక / పత్రికను చదువుతారు<br>(క్లైంట్ నిరక్షరాస్యుడు అయితే దాటవేయండి)		
		Listens to the radio at least once a week<br>వారానికి కనీసం ఒకసారి రేడియోను వింటారు		
		Watches television at least once a week<br>వారానికి కనీసం ఒకసారి టివి చూస్తారు		
		Visits cinema/theatre at least once a month<br>నెలకు కనీసం ఒకసారి సినిమా / థియేటర్‌కి వెళతారు		
**Section B2: Neighbourhood Social Capital and self rated health, happiness and optimism in life**<br>**సెక్షన్ బి2 : చుట్టు ప్రక్కల వారి సమాజిక సంబంధాలు మరియు స్వంతమునకు సంబంధించి జీవితంలో ఆరోగ్యం, సంతోషం మరియు ఆశావాదం**				
B11	Now I will ask you some questions about characteristics of your neighbourhood. Please provide your opinion as Strongly disagree, Disagree, Neither agree nor disagree, Agree or Strongly agree against each statement<br>ఇప్పుడు నేను మీ చుట్టు ప్రక్కలవారి లక్షణాల గురించి కొన్ని ప్రశ్నలు మిమ్ములను అడుగుతాను. దయచేసి ఒక్కో వాక్యముపై ఖచ్చితంగా అంగీకరించను, అంగీకరించను, అంగీకరిస్తాను అనికాదు లేక అంగీకరించను అనికాదు, అంగీకరిస్తాను లేక ఖచ్చితంగా అంగీకరిస్తానుగా మీ అభిప్రాయం తెలుపండి	People say 'hello' and often stop to talk to each other in the street.<br>ప్రజలు హల్లో అని పలుకరిస్తారు మరియు వీధిలో ఒకరినొకరు మాటాలడుటకు తరుచూ ఆగుతారు		**(Code Strongly disagree=5, Disagree=4, Neither agree nor disagree=3, Agree=2, Strongly agree=1)**<br>**(ఖచ్చితంగా అంగీకరించను= 5, అంగీకరించను=4, అంగీకరిస్తానని కాదు లేక అంగీకరించనని కాదు=3, అంగీకరిస్తాను=2, ఖచ్చితంగా అంగీకరిస్తాను=1 గా కోడ్ చేయండి)**
		It is safe for younger children to play outside during the day.<br>పగటి పూట బయట ఆడుకొనుటకు చిన్న పిల్లలకు సురక్షితమైనది		
		You can trust people around here.<br>మీరు ఇక్కడ చుట్టు ప్రక్కల ఉన్నవారిని నమ్మవచ్చు		
		There are good places to spend your free time.<br>మీ ఖాళీ సమయంను గడుపుటకు మంచి చోట్లు ఉన్నవి		
		You can ask for help or a favour from neighbours.<br>మీరు ఇరుగుపొరుగువారి నుండి సహయం లేక అనుకూలంగా చేయమని అడగవచ్చు.		
B12	How would you describe your overall health?<br>మీ మొత్తంమీద ఆరోగ్యముని మీరు ఎలా వర్ణిస్తారు?	Poor బాగాలేదు	1	
		Fair ఫర్వాలేదు	2	
		Good బాగుంది	3	
		Very Good చాలా బాగుంది	4	
		Excellent అద్భుతంగా ఉంది	5	
B13	In general, how do you feel about your life at present?<br>సాధారణంగా,<br>ప్రస్తుతం మీ జీవితం గురించి మీకు ఎలా అనిపిస్తుంది?	I feel very happy<br>నాకు చాలా సంతోషంగా అనిపిస్తుంది	1	
		I feel quite happy<br>నాకు సంతోషంగా అనిపిస్తుంది	2	
		I don't feel very happy<br>నాకు అంత బాగా సంతోషంగా అనిపించుట లేదు	3	
		I am not happy at all.<br>నాకు అస్సలు సంతోషంగా లేదు	4	

Q. No.	Question	Response(s)	Code	Remark<br>DK=99, NR=999
B14.	To what extent have you been bothered by different health problems?<br>విభిన్న ఆరోగ్య సమస్యలచే మీరు ఎంతవరకు బాధపడుతున్నారు?	Not at all అస్సలు లేదు	1	
		A little కొంచెం	2	
		Somewhat కొంతవరకు	3	
		A fair amount చాలా	4	
		A great deal అత్యంత బాగా	5	
B15.	To what extent have these problems affected your life overall?<br>మొత్తంమీద మీ జీవితముని ఈ సమస్యలు ఎంతవరకు ప్రభావం చూపినాయి?	Not at all అస్సలు లేదు	1	
		A little కొంచెం	2	
		Somewhat కొంతవరకు	3	
		A fair amount చాలా	4	
		A great deal అత్యంత బాగా	5	
**Section C1: Tobacco Use information of the Client**<br>**సెక్షన్ సి1: క్లైంట్ యొక్క పొగాకు వాడకపు సమాచారం**				
C01	In what form do you consume tobacco?<br>మీరు ఏ రూపంలో పొగాకు వాడుతారు?	Smoke పొగ రూపంలో	1	
		Smoke less పొగ రహితంగా	2	
		Both రెండూ	3	
C02	How old were you when you first started using tobacco?<br>మీరు పొగాకు వాడుట మొదటిసారిగా ఆరంభించినప్పుడు మీ వయస్సు ఎంత?	Age in completed years ---------<br>వయస్సు పూర్తి అయిన సం॥లలో		Enter age as code<br>పూర్తి అయిన సం॥లలో వయస్సును కోడ్‌గా ఎంటర్ చేయండి
C03	Kindly mention the SMOKING tobacco products you are using with its daily frequency (Enter frequency in the code field)<br>Frequency = Number of sticks / hookah sessions<br>మీరు వాడుతున్న పొగాకు ఉత్పత్తులను త్రాగుటను దాని రోజువారి తరుచుదనంతో తెలుపండి (కోడ్ ఫీల్డ్‌లో తరుచుదనం వ్రాయండి)<br>తరుచుదనం = బీడి/సిగరెట్స్ / సిగార్స్ సంఖ్య లేక రోజువారి హుక్కా సెషన్స్ సంఖ్య	Bidi బీడి		Enter frequency as code<br>తరుచుదనంగా కోడ్‌గా ఎంటర్ చేయండి
		Manufactured Cigarettes<br>ఉత్పత్తి చేసిన సిగరెట్స్		
		Rolled Tobacco in paper or Leaf<br>పేపర్ లేక ఆకులో చుట్టిన పొగాకు		
		Cigars, Cheroots or cigarillos<br>సిగార్స్, చీరూట్స్ లేక సిగారిలోస్		
		Hookah (sessions)<br>హుక్కా (సెషన్స్)		
		Others (Specify)<br>మరేదైనా (తెలుపండి)		
C04	Kindly mention the SMOKELESS tobacco products you are using with its daily frequency of use (Enter frequency in the code field)<br>Frequency = Number of sessions/ times where SMOKELESS tobacco products are being used<br>మీరు వాడుతున్న పొగ రహిత పొగాకు ఉత్పత్తులను త్రాగుటను దాని రోజువారి తరుచుదనంతో తెలుపండి (కోడ్ ఫీల్డ్‌లో తరుచుదనం వ్రాయండి)<br>తరుచుదనం = పొగరహిత పొగాకు ఉత్పత్తులను వాడే రోజువారి తరుచుదనం / సెషన్స్ సంఖ్య	Ghutka or tobacco, lime, areca nut mixture<br>గుట్కా లేక పొగాకు, లైమ్, పక్క మిశ్రమం		Enter frequency as code<br>తరుచుదనంగాను కోడ్‌గా ఎంటర్ చేయండి
		Khaini or Tobacco lime mixture<br>ఖైనీ లేక పొగాకు లైమ్ మిశ్రమం		
		Betel quid with tobacco<br>పొగాకుతో బీటల్ క్విడ్ (పాన్)		
		Panmasala with tobacco<br>పొగాకుతో పాన్‌మసాలా		
		Snuff, Mishri, Gul, Gudakhu<br>నశం, మిశ్రి, గుల్ , గుడాకు		
		Nasal use of snuff<br>నశంను ముక్కుద్వారా వాడకం		
		Others (Specify)<br>మరేదైనా (తెలుపండి)		
C05	What factor(s) made you initiate tobacco use? (Multiple Response possible)<br>మీరు పొగాకు వాడకంను మొదలు పెట్టేలా చేసిన అంశము(అంశాలు) ఏమిటి? (అనేక జవాబులు రావచ్చును)	Peer pressure తోటివారి ఒత్తిడి	1	
		Tobacco users at home ఇంటి వద్ద పొగ త్రాగేవారు	2	
		Style/Fashion statement స్టైల్ / ఫ్యాషన్‌కి నిర్వచనం	3	
		Stress ఒత్తిడి	4	
		Coping హ్యాండిల్ చేయుట	5	
		Experimentation ప్రయోగం చేయుట	6	
		Curiosity ఉత్సుకత	7	
		Others (Specify)<br>మరేదైనా (తెలుపండి)	8	

Q. No.	Question	Response(s)	Code	Remark<br>DK=99, Refused =999
C06	What factors influenced/ supported you to continue tobacco use? (Multiple Response possible)<br>మీరు పొగాకు వాడకంను కొనసాగించుటకు ప్రభావిత ం చేసిన / సహకరించిన అంశాలు ఏమిటి<br>(అనేక జవాబులు రావచ్చును)	Gained financial independence<br>ఆర్థిక స్వేచ్ఛ లభించినది	1	
		Being distant from one's family<br>కుటుంబమునకు దూరంగా ఉండుట	2	
		Tension reduction టెన్షన్ తగ్గించేది	3	
		Relaxation హాయినివ్వటం	4	
		Stimulation ఉత్తేజం కలిగించుట	5	
		Became habit అలవాటుగా కావటం	6	
		To pass time with friend<br>స్నేహితునితో సమయం గడుపుటకు	7	
		Others (Specify)<br>మరేదైనా (తెలుపండి)	8	
C07	Do you use any other habit forming substances other than tobacco?<br>పొగాకు గాక మీరు మరేదైనా అలవాటుగా ఏర్పడిన వాటిని వాడుతారా?	Yes అవును	1	
		No లేదు	2	S » C08
	If YES Select One **అవునంటే ఒకటి ఎంచుకొనండి.**			

Product	Code	Product	Code	
Alcohol ఆల్కహాలు	1	cough syrups దగ్గు టానిక్	8	
Ganja గంజాయి	2	Sleeping tablets నిద్ర కలిగించే బిళ్ళలు	9	
Bhang భంగ్	3	Methadone మెథడోన్	10	
Charas చరాస్	4	Amphetamine యాంఫిటామిన్	11	
Dhatura ధాతురా	5	Brown sugar బ్రౌన్ షుగర్	12	
Opium ఒపియమ్	6	Smack స్మాక్	13	
Heroine హెరాయిన్	7	Other (specify) మరేదైనా (తెలుపండి)	14	

MINI QUESTIONNAIRE TO ASSESS TOBACCO DEPENDENCE AS PER ICD -10 & DSM-IV
[Ask following (C08 TO C14) questions with reference to last 12 months only]
(క్రింద ప్రశ్నలను (C08 TO C14) గత 12 నెలల కాలంలో పొగాకు వాడకం గురించి)

Q. No.	Question	Code	Remark
C08	Have you found that you needed to consume much more (NAME OF TOBACCO PRODUCT) to get the same effect that you did when you first started taking it?<br>మీరు మొదటిసారిగా **(పొగాకు ఉత్పత్తి పేరు)** ని తీసుకొనుట మొదలు పెట్టినప్పుడు మీరు చేసిన అదే ప్రభావమును పొందుటకు మీరు దానిని చాలా ఎక్కువ మొత్తం వాడాల్సిన అవసరం ఉందని మీకు అనిపించినదా?		(Yes=1, No=2)<br>(అవును=1, లేదు=2)
C09	Have you often found that when you consumed (NAME OF TOBACCO PRODUCT), you ended up taking more than you thought you would take?<br>మీరు (పొగాకు ఉత్పత్తి పేరు)ని వాడినప్పుడు, మీరు అనుకొన్న దాని కంటే ఎక్కువ సమయం పాటు కొనసాగించినారని మీకు ఎంత తరుచుగా అనిపిస్తుంది?		(Yes=1, No=2)<br>(అవును=1, లేదు=2)
C10	a. Have you tried to reduce or quit (NAME OF TOBACCO PRODUCT)?.<br>మీరు (పొగాకు ఉత్పత్తి పేరు) తగ్గించుటకు లేక మానివేయుటకు ప్రయత్నించినారు? , కాని విఫలం అయినారా?		(Yes=1, No=2)<br>(అవును=1, లేదు=2) If NO Skip to C11<br>లేదు అంటే C11కి వెళ్ళండి
	b. If YES, did you fail or succeed to reduce/quit?<br>అవును అంటే, తగ్గించుట / మానివేయుట లో విఫలం లేదా సఫలం అయినారా?		(Fail=1, Succeed=2)<br>(అవును=1, లేదు=2)
C11	On the days that you used (NAME OF TOBACCO PRODUCT), did you spend substantial time (>2 HOURS),<br>Code (Yes=1, No=2) against each of the FOLLOWING options<br>మీరు **(పొగాకు ఉత్పత్తి పేరు)** వాడిన రోజులలో, తగినంత సమయం గడిపినారా (>2గంటలు)?<br>క్రింద ప్రశ్నలకు (అవును=1, లేదు=2) అని కోడ్ చేయండి		
	A) OBTAINING and USING tobacco product<br>పొగాకు ఉత్పత్తి వాడుట మరియు పొందుటలో		
	B) In recovering from tobacco product use<br>పొగాకు ఉత్పత్తి వాడకం నుండి కోలుకొనుట		(Yes=1, No=2)<br>(అవును=1, లేదు=2)
	C) In thinking about the tobacco product<br>పొగాకు ఉత్పత్తి గురించి ఆలోచించుట		

Q. No.	Question	Response(s)	Code	Remark<br>DK=99, Refused =999
C12	Did you spend less time working, enjoying hobbies, or being with family or friends because of your tobacco use?<br>మీరు పొగాకు వాడటం వలన పని చేయుట, హాబిలపై ఎంజాయ్ చేయుట లేక, కుటుంబము లేక స్నేహితులతో తక్కువ సమయం గడిపినారా?			(Yes=1, No=2)<br>(అవును=1, లేదు=2)
C13	If, tobacco caused health/mental problems, did you still keep on using it?<br>పొగాకు మీ ఆరోగ్య / మానిసక సమస్యలు కలిగించినప్పటికీ, మీరు దానిని ఇంకా వాడుతూ ఉన్నారా?			(Yes=1, No=2, 3= tobacco has not caused any such problem)<br>(అవును=1, లేదు=2, 3=పొగాకు ఎలాంటి సమస్యలు కలిగించ లేదు
C14	A) When you reduced or stopped using (NAME OF TOBACCO PRODUCT) did you face any withdrawal symptoms (aches, shaking, fever, weakness, diarrhea, nausea, sweating, heart pounding, difficulty sleeping, or feeling agitated, anxious, irritable, or depressed)? (CODE Yes=1, No=2)<br>మీరు (**పొగాకు ఉత్పత్తి పేరు)** ని వాడుట తగ్గించినప్పుడు లేక మానివేసినప్పుడు, మీకు ఉపసంహారణ లక్షణాలు (నొప్పులు, వణుకు, జ్వరం, నీరసం, అతిసారం, వికారం, చెమట పట్టడం, గుండె నొప్పి, నిద్ర పట్టడంలో ఇబ్బంది లేక అసహనంగా అనిపించుట, ఆందోళన, చికాకుగా లేక కృంగిపోయినట్లుగా) కలిగినాయా? (అవును=1, లేదు=2 అని కోడ్)			(Yes=1, No=2)<br>(అవును=1, లేదు=2)
	B) Did you use any tobacco products to keep yourself away from getting sick (withdrawal symptoms), or so that you would feel better? (CODE Yes=1, No=2)<br>అనారోగ్య పడుట (**మానివేసినందు వలన లక్షణాలు**) నుండి మిమ్ములను కాపాడుకొనుటకు మీరు ఏదైనా పొగాకు ఉత్పత్తులను వాడినారా లేక ఆవిధంగా మీకు బాగా అనిపిస్తుంది? (అవును=1, లేదు=2 అని కోడ్)			(Yes=1, No=2)<br>(అవును=1, లేదు=2)
C15	What is your overall opinion of tobacco use?' Code as ***(very good=5, good=4, neither good nor bad=3, bad=2, very bad=1)***<br>పొగాకు వాడకంపై మొత్తంమీద మీ అభిప్రాయం ఏమిటి? (చాలా బాగుంది=5, బాగుంది=4, బాగుందని కాదు లేక బాగాలేదని కాదు=3, బాగాలేదు=2, చాలా బాగాలేదు=1)			
	**Fagerstrom's Test for Nicotine Dependence (FTND)**			
C16	**FTND for Smoking Tobacco User**		Score	
**Smoking Tobacco User**	1. How soon after you wake up, do you smoke your first bidi/cigarette?<br>మీరు నిద్ర లేచిన తరువాత, మీరు మొదటిగా బీడి / సిగరెట్ ఎంత త్వరగా త్రాగుతారు?	Within 5 minutes<br>5 నిముషాల లోపు	3	
		6-30 minutes నిముషాలు	2	
		31-60 minutes<br>నిముషాలు	1	
		After 60 minutes<br>నిముషాలు తరువాత	0	
	2. Do you find it difficult to refrain from smoking in places where it is forbidden (e.g. at the library, cinema, park, restaurant, public transports etc.)?<br>నిషేధించబడిన చోట్లలో పొగ త్రాగుట నుండి అదుపు చేసుకొనుట మీకు కష్టంగా అనిపిస్తుందా (ఉదా: లైబ్రరీ, సినిమా, పార్క్, రెస్టారెంట్, పబ్లిక్ ప్రయాణ సాధనాలు మొ)	Yes	1	
		No	0	
	3. Which cigarette would you hate to give up?<br>మీరు ఏ సిగరెట్ మానివేయుటకు అసహ్యించు కొంటారు?	The first one in the morning<br>ఉదయం పూట మొదటిది	1	
		All the others<br>మిగతా అన్ని	0	Q4 of FTND is covered in C03
	5. Do you smoke more frequently during the first hours after waking than during the rest of the day?<br>రోజులో మిగతా సమయం కంటే ఉదయం నిద్ర లేచిన తరువాత మొదటి గంటలలో మీరు ఎక్కువ తరుచుగా పొగ త్రాగుతారా?	Yes	1	
		No	0	
	6. Do you smoke if you are so ill, that you are in bed most of the day?<br>మీరు రోజులో ఎక్కువ సేపు మంచం మీద ఉండేలా మీరు బాగా అనారోగ్యంగా ఉంటే మీరు పొగ త్రాగుతారా?	Yes	1	
		No	0	

Q. No.	Question	Response(s)	Code	Remark<br>DK=99, Refused =999
C17	**FTND for Smokeless Tobacco User**<br>పొగ రహిత పొగాకు వాడేవారు		Score	
Smokeless Tobacco User	1. How soon after you wake up do you place your first quid (chew)?<br>మీరు నిద్ర లేచిన తరువాత ఎంత త్వరగా<br>మీ మొదటి పొగాకు **ఉత్పత్తి** పెట్టుకొంటారు?	Within 5 minutes 5 నిముషాల లోపు	3	Q4 of FTND is covered in C04
		6-30 minutes నిముషాలు	2	
		31-60 minutes నిముషాలు	1	
		After 60 minutes నిముషాలు తరువాత	0	
	2. How often do you intentionally swallow tobacco juice?<br>మీరు కావాలని ఎంత తరుచుగా పొగాకు రసంని వి ంగుతారు?	Always ఎల్లప్పుడూ	2	
		Sometimes కొన్నిసార్లు	1	
		Never ఎన్నడూ లేదు	0	
	3. Which chew would you hate to give up most?<br>ఏది నములుటను మానివేయుటకు అత్యధికంగా అస ్టలు ఇష్టపడరు?	The first one in the morning<br>ఉదయం పూట మొదటిది	1	
		All the others<br>మిగతా అన్ని	0	
	5. Do you chew more frequently during the first hours after awakening than during the rest of the day?<br>రోజులో మిగతా సమయం కంటే ఉదయం<br>నిద్ర లేచిన తరువాత మొదటి గంటలలో<br>మీరు ఎక్కువ తరుచుగా నములుతారా?	Yes	1	
		No	0	
	6. Do you chew if you are so ill, that you are in bed most of the day??<br>మీరు రోజులో ఎక్కువ సేపు అనారోగ్యంగా ఉండి మ ంచం మీద ఉన్నా మీరు నములుతారా?	Yes	1	
		No	0	
C18.	How many times have you tried to quit tobacco use during the past 12 months?<br>గడిచిన 12 నెలల్లో పొగాకు వాడకమును<br>మానివేయుటకు మీరు ఎన్నిసార్లు ప్రయత్నించినారు?	0	1	వాడకము మానివేయుటకు ప్రయత్ని ంచిక పోతే If nil quit attempt S » C22
		1 to 5	2	
		5 to 10	3	
		>10	4	
C19	How long ago did your most recent serious quit attempt end? or last?<br>మీ చిట్టచివరి తీవ్రంగా పొగ మానివేసే యత్నం<br>ఎంత కాలం క్రితం జరిగింది?	6 or more months లేక ఎక్కువ నెలలు	1	
		< 6 months 6 నెలల కంటే ఎక్కువ	2	
		1-6 months నెలలు	3	
		Weeks వారాలు	4	
		Days రోజులు	5	
		Less than 24 hours<br>24 గంటల కంటే తక్కువ	6	
C20	What was the motivational factor for your last quitting attempt? Code as (1= not at all, 2 = a little, 3=very much)<br>మీ చివరిసారి మానివేసే ప్రయత్నంకు ప్రోత్సహించే అంశము ఏది? (1=అస్సలు లేదు, 2= కొంచెం, 3=చాలా ఎక్కువ)<br><br>**READ OUT EACH RESPONSE AND CODE.**<br>**MULTIPLE RESPONSE POSSIBLE**<br>(అనేక జవాబులు రావచ్చును)	Concern for your personal health?<br>మీ వ్యక్తిగత ఆరోగ్యమునకు ఆందోళన?		
		Concern about the effect of your smoking on non-smokers?<br>పొగ త్రాగనివారిపై మీరు పొగ త్రాగుట వలన ప్రభావం<br>గురించి ఆందోళన		
		That society disapproves of smoking<br>పొగ త్రాగుటను సమాజం ఆమోదించకపోవు ట		
		The price of cigarettes/bidis<br>సిగరెట్స్ / బీడిల యొక్క ధర		
		Smoking restrictions in public and work places<br>పబ్లిక్ మరియు పని చేసే ప్రదేశాలలో పొగ త్రాగుటపై నిబంధనలు		

Q. No.	Question	Response(s)	Code	Remark DK=99, Refused =999
		Advertisements or information about the health risks of smoking పొగ త్రాగుట వలన ఆరోగ్య ప్రమాదాల గురిం చి ప్రకటనలు లేక సమాచారం		
		Health warning labels on cigarette packages? సిగరెట్ ప్యాకేజిలపై ఆరోగ్య హెచ్చరిక లేబుల్స్ ?		
		Setting an example for children? పిల్లలకు ఉదాహరణ నెలకొల్పుట?		
		Your family disapproves of smoking? పొగ త్రాగుటను మీ కుటుంబము ఆమోదించ కపోవుట?		
		OTHERS (Specify)…………		
C21	During the past 12 months, did you use any of the following to try to quit tobacco? IF YES CODE=1, IF NO CODE=2 గడిచిన 12 నెలల్లో, మీరు పొగాకు మానివేయుటకు వీటిలో ఏవైనా వాడినారా? READ OUT AND CODE EACH ANSWER MULTIPLE RESPONSE POSSIBLE (అనేక జవాబులు రావచ్చును)	Counseling, including at a smoking cessation clinic? కౌన్సిలింగ్,పొగ త్రాగుటని మాన్పించు క్లినిక్ వ ద్ద కలుపుకొని		
		Nicotine replacement therapy, such as the gum నికోటిన్ మార్చివేసే థెరపీ, అంటా గమ్ లాం టిది		
		Other prescription medications, for example Bupropion? ఇతర ప్రిస్క్రిప్షన్ మందులు ఉదా: బ్యూప్రోపియ ఎన్		
		Traditional medicines, for example Ayurvedic, Homeopathic, Unani? సాంప్రదాయ మందులు ఉదా : ఆయుర్వేదిక్, హోమియోపతిక్, యునాని మొ.		
		Cessation or an anti tobacco telephone support line? ముగించే లేక యాంటీ పొగాకు టెలిఫోన్ సపో ర్ట్ లైన్?		
		Switching to smokeless tobacco? పొగ రహిత పొగాకుకి మారుట?		
		Anything else? Specify: ఇంకా ఏమైనా? తెలుపండి :		
C22	Which of the following best describes the practices about smoking inside of your home మీ ఇంటిలోపల పొగ త్రాగుట గురించి అలవాట్లను వీటిలో ఏది బాగా తెలుపుతుంది?	Smoking is allowed inside of your home మీ ఇంటిలోపల పొగ త్రాగుట అనుమతించబ డుతుంది	1	
		smoking is generally not allowed inside of your home but there are exceptions, సాధారణంగా మీ ఇంటిలోపల పొగ త్రాగుట అనుమతించబడదు, కాని కొన్ని మినహాయింప ులు ఉన్నాయి	2	
		smoking is never allowed inside of your home మీ ఇంటిలోపల పొగ త్రాగుటకు ఎన్నడూ అనుమ తించబడదు	3	

Q. No.	Question	Response(s)	Code	Remark DK=99, Refused =999
C23	How often does anyone smoke inside your home? Would you say daily, weekly, monthly, less than monthly, or never? మీ ఇంటిలోపల ఎవరైనా ఎంత తరుచుగా పొగ త్రాగుతారు? అది రోజూ, వారానికోసారి, నెలకోసారి, నెలకోసారి కంటే తక్కువ తరుచుగా లేక ఎన్నడూ త్రాగరు అని మీరు అంటారా?	Daily రోజూ	1	
		Weekly వారానికోసారి	2	
		Monthly నెలకోసారి	3	
		Less than monthly నెలకు ఒకసారి కంటే తక్కువగా	4	
		Never ఎన్నడూ లేదు	5	
C24	Did anyone smoke inside of any health care facilities DURING your today's visit? మీరు ఈరోజున వచ్చిన సమయంలో ఏదైనా ఆరోగ్య కేంద్రాల లోపల ఎవరైనా పొగ త్రాగినారా?	Yes	1	
		No	2	
C25	Did anyone smoke inside of any health care facilities that you visited in the past 30 days? గడిచిన 30 రోజులలో మీరు వెళ్ళిన ఏదైనా ఆరోగ్య సంరక్షణ కేంద్రాలలో ఎవరైనా త్రాగినారా?	Yes	1	
		No	2	
C26	Have you noticed any IEC material advocating quitting tobacco in the health facility during this visit? ఈ సందర్భనలో ఆరోగ్య కేంద్రంలో పొగాకు మానివేయుటకు సలహాలిచ్చే ఏదైనా ఐఇసి మెటీరియల్ని మీరు గమనించినారా?	Yes అవును	1	
		No లేదు	2	
**SECTION D: TOBACCO COUNSELLING PRACTICES BY HEALTH CARE SERVICE PROVIDERS** **సెక్షన్ డి : పొగాకు వాడకంపై ఆరోగ్య సంరక్షణ సేవలనందించేవారిచే కౌన్సిలింగ్ పద్ధతులు**				
D01	How many times did you visit a doctor or health care provider in the past 12 months? గడిచిన 12 నెలల్లో మీరు డాక్టరు లేక ఆరోగ్య సంరక్షణ అందించేవారి వద్దకు ఎన్నిసార్లు వెళ్ళినారు?	1 or 2 TIMES 1 లేక 2 సార్లు	1	
		3 to 5 TIMES 3 నుండి 5 సార్లు	2	
		6 or more TIMES 6 లేక ఎక్కువ సార్లు	3	
D02	For what condition/health concern did you visit this health facility?(Refer to OPD Slip if necessary) మీరు ఈ ఆరోగ్య కేంద్రమునకు ఏ పరిస్థితి / ఆరోగ్య సమస్య గురించి వచ్చినారు? (అవసరమైతే ఒపిడి స్లిప్ చూడండి)	Cough & other Respiratory problems దగ్గు మరియు ఇతర శ్వాస సంబంధ సమస్యలు	1	
		General ailments (fever, wounds, Diarrhea etc.) సాధారణ అనారోగ్యములు (జ్వరం, గాయాలు, అతిసారం)	2	
		**ANC** కడుపుతో ఉన్నప్పుడు పరీక్షలు	3	
		Delivery కాన్పు	4	
		Family Planning కుటుంబ నియంత్రణ	5	
		Immunization రోగనిరోధక టీకాలు	6	
		RTI/STI సుఖవ్యాధులు	7	
		**TB** క్షయ వ్యాధి	**8**	
		Communicable diseases అంటువ్యాధులు	9	
		Trauma ట్రూమా	10	
		Chronic conditions (CVD – Heart Diseases, Asthma, Diabetics, Cancers) దీర్ఘకాలిక పరిస్థితులు (సివిడి–గుండె వ్యాధులు, ఉబ్బసం, మధుమేహం, క్యాన్సర్, మొ)	11	
		Others , Specify_____________ మరేదైనా, తెలుపండి	12	

Q. No.	Question	Response(s)	Code	Remark<br>DK=99, Refused =999
D03	Who did you see for your condition/ health concern?<br>మీ పరిస్థితి / ఆరోగ్య సమస్య గురించి ఎవరి వద్దకు వెళ్ళినారు?	Medical Officer మెడికల్ అధికారి	1	
		Specialist Specify (---------)<br>స్పెషలిస్ట్ (తెలుపండి)	2	
		Pharmacist ఫార్మసిస్ట్	3	
		Others Specify (------------)<br>ఇతరులు తెలుపండి	4	
D04	Have you been asked about your tobacco consumption habit during today's visit?<br>ఈరోజున వెళ్ళిన సమయంలో మీ పొగాకు వా డకం<br>గురించి మిమ్ములను అడిగినారా?	Yes, during this visit<br>అవును, ఈ సందర్శనలో	1	
		Not during this visit, but during earlier visit<br>ఈ సందర్శనలో కాదు, కాని **గత** సందర్శనలో	2	Skip to Section E<br>సెక్షన్ E కి వెళ్ళండి
		Never, in any of the visits<br>లేదు, ఏ సందర్శనలోను	3	
D05	When was your tobacco consumption history asked?<br>మీ పొగాకు వాడకపు చరిత్రను ఎప్పుడు అడిగి నారు?	During registration రిజిస్ట్రేషన్ సమయంలో	1	
		During general history taking<br>సాధారణ చరిత్ర అడిగే సమయంలో	2	
		During prescribing medicine<br>మందులను సూచించే సమయంలో	3	
		Other (Specify)<br>మరేదైనా (తెలుపండి)	4	
D06	Did your health care provider advice/counsel you on ways to quit tobacco use during your visit?<br>మీసందర్శనలో పొగాకు మానివేయుటకు మా ర్గాలపై మీ ఆరోగ్య సంరక్షణను అందించేవారు మీకు సలహా / కౌన్సిల్ చేసినారా?	Yes, during this visit<br>అవును, ఈ సందర్శనలో	1	
		Not during this visit, but during earlier visit<br>ఈ సందర్శనలో కాదు, కాని **గత** సందర్శనలో	2	Skip to Section E<br>సెక్షన్ E కి వెళ్ళండి
		Never, in any of the visits<br>లేదు, ఏ సందర్శనలోను	3	
D07	How long did the discussion / advice / counseling on quitting tobacco use last?<br>పొగాకు వాడకం మానివేయుటపై చర్చ / సలహా / కౌన్సిలింగ్ ఎంత సేపు ఉన్నది?	< 30 sec 30 సెకండ్ల లోపు	1	
		30 Sec to 1 min 30 సెకండ్ల నుండి 1 నిముషం	2	
		1-5 min నిముషాలు	3	
		6 min or more నిముషాలు లేక ఎక్కువ	4	
D8	During your interaction with health provider today, did the provider<br>ఈరోజున డాక్టరుతో మీ సంభాషణ సమయంలో డాక్టరు .....?			
	a. Give information on harmful effects of tobacco?<br>పొగాకు వలన హానికరమైన ప్రభావాలపై సమాచారం ఇచ్చినారా?	Yes అవును	1	
		No లేదు	2	
	b. Give information on benefits of quitting tobacco?<br>పొగాకు మానివేయుట వలన ప్రయోజనాలపై సమాచారం ఇచ్చినారు	Yes అవును	1	
		No లేదు	2	
	c. Asked about your intention/ interest to quit<br>మానివేయుటకు మీ ఆసక్తి గురించి అడిగినారు	Yes అవును	1	
		No లేదు	2	
	d. Asked for your willingness to quit<br>మానివేయుటకు మీ ఇష్టమును అడిగినారు	Yes అవును	1	
		No లేదు	2	
	e. Suggest ways to quit tobacco use<br>పొగాకు మానివేయుటకు మార్గాలను సూచించుట	Yes అవును	1	
		No లేదు	2	
	f. Suggest quitting tobacco but did not mention the ways to quit<br>పొగాకు మానివేయుటకు సూచించినారు, కాని మానివేసే మార్గాలను తెలుపలేదు.	Yes అవును	1	
		No లేదు	2	
	g. Informed you about different medicines for quitting<br>మానివేయుటకు విభిన్న మందుల గురించి మీకు తెలిపినారు	Yes అవును	1	
		No లేదు	2	
	h. Informed you about the further follow up at higher centers?<br>ఉన్నత కేంద్రాల వద్ద తరువాత ఫాలోఅప్ గురించి మీకు తెలిపినారు?	Yes అవును	1	
		No లేదు	2	

Q. No.	Question	Response(s)	Code	Remark DK=99, Refused =999
	i. Tell you when to return for follow-up counseling<br>ఫాలో అప్ కౌన్సిలింగ్‌కి మరలా ఎప్పుడు రావాలో మీతో చెప్పినారు	Yes అవును	1	
		No లేదు	2	
	j. Explain your present health condition as a result of your tobacco use<br>మీ పొగాకు వాడకం ఫలితంగా మీ ప్రస్తుత ఆరోగ్య పరిస్థితిని వివరించినారు.	Yes అవును	1	
		No లేదు	2	
	k. Explain the benefits of quitting tobacco use w.r.t. your health condition<br>మీ ఆరోగ్య పరిస్థితికి సంబంధించి పొగాకు వాడకం మానివేయుట లో ప్రయోజనాలను వివరించుట	Yes అవును	1	
		No లేదు	2	
	l. Address the difficulties you are facing in quitting tobacco<br>పొగాకు మానివేయుటలో మీరు ఎదుర్కొనే ఇబ్బందులను తీర్చుట	Yes అవును	1	
		No లేదు	2	
D9	Did you feel comfortable during counseling for quitting?<br>మానివేయుటకు కౌన్సిలింగ్ సమయంలో మీకు సౌకర్యవంతంగా అనిపించినదా?	Not at all comfortable<br>అస్సలు సౌకర్యవంతంగా లేదు	1	
		Somewhat comfortable<br>కొంతవరకు సౌకర్యవంతంగా ఉంది	2	
		Very comfortable<br>చాలా సౌకర్యవంతంగా ఉంది	3	
D10	How would you describe your overall satisfaction with the service you received?<br>మీకు అందిన సర్వీస్‌తో మీరు మొత్తంమీద సంతృప్తిని మీరు ఎలా వివరిస్తారు?	Satisfied సంతృప్తి చెందినారు	1	
		Not Satisfied<br>సంతృప్తి చెందలేదు	2	
		Uncertain ఖచ్చితంగా చెప్పలేను	3	
D11	Are you planning to reduce tobacco use in the near future after listening to the advice / counseling?<br>సలహా / కౌన్సిలింగ్ విన్న తరువాత మీరు సమీప భవిష్యత్తులో పొగాకు వాడకం తగ్గించాలనుకుంటున్నారా?	Yes అవును	1	
		No లేదు	2	
D12	Are you planning to quit tobacco in the near future after listening to the advice/counseling?<br>సలహా / కౌన్సిలింగ్‌ని విన్న తరువాత సమీప భవిష్యత్తులో మీరు పొగాకు మానివేయాలనుకొంటున్నారా?	Yes అవును	1	
		No లేదు	2	
D13	Have you decided upon a quit date during the counselling?<br>కౌన్సిలింగ్ సమయంలో మానివేయాల్సిన తేదిపై మీరు నిర్ణయించు కొన్నారా?	Yes అవును	1	
		No లేదు	2	
D14	Do you feel counselling/advice would help in reducing /quitting tobacco use?<br>పొగాకు వాడకం మానివేయుట / తగ్గించుకొనుటలో కౌన్సిలింగ్/ సలహా సహాయపడుతుందని మీకు అనిపిస్తుందా?	Strongly agree<br>ఖచ్చితంగా అంగీకరిస్తాను	1	
		Agree అంగీకరిస్తాను	2	
		Neither Agree nor disagree<br>అంగీకరిస్తాను అనికాదు లేక అంగీ కరించను అనికాదు	3	
		Disagree అంగీకరించను	4	
		Strongly disagree<br>ఖచ్చితంగా అంగీకరించను	5	
D15	Would you recommend other tobacco users to get counseled by doctors for quitting tobacco?<br>మీరు ఇతర పొగాకు వాడకందార్లకు పొగాకు మానివేయుటకు డాక్టర్‌చే కౌన్సిలింగ్ చేయించుకోమని సిఫారస్ చేస్తారా?	Yes అవును	1	
		No లేదు	2	

Section E: Motivation to Quit and Tobacco Cessation			
.	**Assessment of Motivation for Quitting (10 Point Scale - Quitting ladder) for tobacco users**<br>పొగాకు వాడకందార్లకు (10 పాయింట్ స్కేలు మానివేసే నిచ్చెన) మానివేయుటకు ప్రోత్సాహముని నిర్ధారణ<br>**READ OUT EACH OPTION AND CODE ONE RESPONSE ONLY (The most appropriate you feel)**		
	I have quit tobacco. నేను పొగాకు మానివేసాను	10	
	I have quit tobacco, but I still worry about slipping back, so I need to keep working on living tobacco free.<br>నేను ఖచ్చితంగా మానివేయాలనుకొంటున్నాను, కాని మరలా మొదలు పెడతామోనని ఇంకా ఆందోళన చెందుతున్నాను, అందుచేత పొగాకు రహితంగా జీవించుటపై నేను ఇంకా పని చేస్తూ ఉండాల్సియుంది	9	
	I still use tobacco, but I have begun to change, like cutting back on the number of cigarettes/bidi I smoke (pouch/can of khaini/gutkha I use). I am ready to set a quit date.<br>నేను ఇంకా పొగాకు వాడుతూ ఉన్నాను, కాని మారటం మొదలు పెట్టినానను, అంతే నేను త్రాగే సిగరెట్స్ / బీడిల (నేను వాడే ఖైనీ / గుట్కా యొక్క పౌచ్ / క్యాన్) సంఖ్య తగ్గించుట లాంటిది. నేను మానివేయుటకు ఇప్పటికే ఒక తేదిని పెట్టుకొన్నాను	8	
	I definitely plan to quit in the next 30 days.<br>వచ్చే 30 రోజులలో నేను ఖచ్చితంగా మానివేయాలనుకొంటున్నాను	7	
	I definitely plan to quit in the next 6 months.<br>వచ్చే 6 నెలల్లో నేను ఖచ్చితంగా మానివేయాలనుకొంటున్నాను	6	
	I often think about quitting, but I have no plans to quit.<br>నేను మానివేయుట గురించి తరుచుగా ఆలోచిస్తున్నాను, కాని మానివేయుటకు నాకు ప్లాన్స్ లేవు	5	
	I sometimes think about quitting, but I have no plans to quit.<br>మానివేయుట గురించి నేను కొన్నిసార్లు ఆలోచిస్తాను, కాని మానివేయుటకు నాకు ప్లాన్స్ లేవు.	4	
	I rarely think about quitting, and I have no plans to quit.<br>మానివేయుట గురించి నేను అరుదుగా ఆలోచిస్తాను, మరియు మానివేయుటకు నాకు ఏ ప్లాన్స్ లేవు	3	
	I never think about quitting, and I have no plans to quit.<br>మానివేయుట గురించి నేను ఎన్నడూ ఆలోచించను, మరియు మానివేయుటకు నాకు ఏ ప్లాన్స్ లేవు	2	
	I have decided not to quit for my lifetime. I have no interest in quitting<br>నా జీవిత కాలం పాటు మానివేయవద్దని నేను నిర్ణయించుకొన్నాను, మానివేయుటలో నాకు ఏ ఆసక్తి లేదు	1	

THANK YOU FOR YOUR PATIENCE AND VALUABLE TIME

[Counsel the Patient for quitting tobacco and provide a take home message after counselling]
